# Plasma Proteomic Profile of Patients with Tick-Borne Encephalitis and Co-Infections

**DOI:** 10.3390/ijms23084374

**Published:** 2022-04-15

**Authors:** Agnieszka Gęgotek, Anna Moniuszko-Malinowska, Monika Groth, Sławomir Pancewicz, Piotr Czupryna, Justyna Dunaj, Sinemyiz Atalay, Piotr Radziwon, Elżbieta Skrzydlewska

**Affiliations:** 1 Department of Analytical Chemistry, Medical University of Bialystok, Mickiewicza 2D, 15-222 Bialystok, Poland; sinemyiz.atalay@umb.edu.pl (S.A.); elzbieta.skrzydlewska@umb.edu.pl (E.S.); 2 Department of Infectious Diseases and Neuroinfections, Medical University of Bialystok, Zurawia 14, 15-540 Bialystok, Poland; anna.moniuszko@umb.edu.pl (A.M.-M.); monika.groth@umb.edu.pl (M.G.); spancewicz@interia.pl (S.P.); avalon-5@wp.pl (P.C.); dunaj.justyna@wp.pl (J.D.); 3 Regional Centre for Transfusion Medicine, M. Sklodowskiej-Curie 23, 15-950 Bialystok, Poland; piotr.radziwon@umb.edu.pl

**Keywords:** tick-borne encephalitis, plasma, proteomic profile, protein adducts, co-infections, Lyme disease, neuroborreliosis, human granulocytic anaplasmosis

## Abstract

Despite the increasing number of patients suffering from tick-borne encephalitis (TBE), Lyme disease, and their co-infection, the mechanisms of the development of these diseases and their effects on the human body are still unknown. Therefore, the aim of this study was to evaluate the changes in the proteomic profile of human plasma induced by the development of TBE and to compare it with changes in TBE patients co-infected with other tick-borne pathogens. The results obtained by proteomic analysis using a nanoLC-Q Exactive HF mass spectrometer showed that the most highly elevated groups of proteins in the plasma of TBE patients with co-infection were involved in the pro-inflammatory response and protein degradation, while the antioxidant proteins and factors responsible for protein biosynthesis were mainly downregulated. These results were accompanied by enhanced GSH- and 4-HNE-protein adducts formation, observed in TBE and co-infected patients at a higher level than in the case of patients with only TBE. In conclusion, the differences in the proteomic profiles between patients with TBE and co-infected patients indicate that these diseases are significantly diverse and, consequently, require different treatment, which is particularly important for further research, including the development of novel diagnostics tools.

## 1. Introduction

It is estimated that the tick population in Europe, Asia, and America is increasing every year. The reasons for this phenomenon are found both in climate change, leading to higher temperatures in winter, as well as in the cessation of mass grass burning in spring or even the large migration of host mammals [1]. Regardless of the species or stage of the life cycle, ticks need the blood of vertebrates to live and reproduce; however, their bites may carry a risk of spreading pathogenic agents. The increase in the geographical range of ticks causes an increasing number of cases of tick-borne diseases, because ticks can transmit bacterial, parasitic, and viral pathogens and often harbor more than one agent simultaneously [2]. Tick-borne diseases are often the cause of long-lasting and bothersome symptoms, permanent health problems, and even death. One of the most common and most dangerous tick-borne diseases, caused by a virus of the genus *Flavivirus*, is tick-borne encephalitis (TBE) [3]. TBE is endemic in focal areas of Europe, Siberia, far-eastern Russia, northern China, and Japan. Over the past few decades, endemic regions have expanded, and the number of cases reported continues to increase. Each year, approximately 5000–13,000 TBE cases are reported, with large annual fluctuations. These numbers are likely underestimated because in many countries, the notification of the disease is not mandatory [4,5]. Because the TBE virus is in the tick’s saliva, it needs little time from the moment of the tick’s bite to reach the human body. The initial symptoms of the disease are nonspecific and include headache, fever, muscle pain, and fatigue. However, as many as 50% of patients with TBE have long-term sequelae. The most frequently reported symptoms include cognitive disturbances, neuropsychiatric problems, headache, hearing loss and/or tinnitus, disturbances of vision, balance and coordination disorders associated with cerebellar syndrome, and flaccid paresis or paralysis [5,6]. About one-third of the patients develop the second phase of the disease, which is characterized by central nervous system involvement and most commonly presents as meningitis and, in more severe cases, meningoencephalitis or meningoencephalomyelitis. The clinical symptoms of meningitis include headache, fever, nausea, vomiting, vertigo, and nuchal rigidity. In meningoencephalitis, in addition to previously mentioned symptoms, patients have impaired consciousness or focal neurological deficits, such as nerve paresis or cerebellar ataxia. Meningoencephalomyelitis is a condition that involves the inflammation of the meninges, brain, and spinal cord, which can present as polio-like flaccid paresis and autonomic dysfunction [7].

TBE can be effectively prevented with vaccination. However, to date, there is no specific antiviral treatment available for TBE. Only symptomatic therapy is possible, which involves the administration of antipyretics, analgesics, antiemetics, and antiedemic treatment and the maintenance of water and electrolyte balance. Corticosteroids are used in especially severe cases; however, their usage remains controversial, as it has been associated with a prolonged duration of hospitalization in some studies. Although corticosteroid administration can appear effective in certain cases, it is not recommended as a standard treatment approach [5,8].

Tick-borne diseases caused by bacteria can also be dangerous for human health and life. The most frequently diagnosed bacterial tick-borne disease is Lyme disease (LD), caused by the bacterium *Borrelia burgdorferi* [3]. The incidence of LD is increasing, and the disease has become a significant public concern across the Northern Hemisphere. An estimated ≈476,000 cases are diagnosed and treated per year in the United States and >200,000 cases per year in western Europe [9]. LD may affect the skin, joints, heart, eyes, and nervous system. While most cases of Lyme disease can be cured with a 2- to 4-week course of antibiotic treatment, some individuals experience health problems that persist for months or even years. This condition is called Post-Treatment Lyme Disease Syndrome (PTLDS) with unspecific symptoms, including widespread musculoskeletal pain, fatigue, or cognitive difficulties lasting for more than 6 months after treatment. The cause of nonspecific symptoms persisting after treatment for Lyme disease remains unclear [10]. The neurological form of LD is called neuroborreliosis (NB) and most commonly presents as facial nerve and other cranial nerves palsy, meningitis, and radiculitis [11].

Human granulocytic anaplasmosis (HGA) is another bacterial tick-borne disease that is recently being increasingly recognized. *Anaplasma phagocytophilum,* the etiologic factor of HGA, is a facultative intracellular bacteria that spreads through the blood and lymph vessels and attacks white blood cells, which infiltrate and spread the infection to the liver, spleen, lymph nodes, kidneys, lungs, and central nervous system. Consequently, HGA causes a wide range of clinical symptoms varying from asymptomatic infection to multisystem organ failure. Patients with HGA most frequently present with fever, sweating, rigors, headache, myalgia, and arthralgia. The typical laboratory findings in *A. phagocytophilum* infection include increased aminotransferases activity, leukopenia, and thrombocytopenia [12].

Patients with severe symptoms of tick-borne diseases require hospitalization; however, why some of them suffer and even die from infection, while others are left with only minor ailments after infection, remains to be discovered. So far, a large amount of research has been carried out in terms of changes in the human body caused by the described pathogens. Some of them clearly indicate oxidative stress and, possibly, activation of the antioxidant system, including stimulation of the NF-E2–related factor 2/antioxidant responsive element (Nrf2/ARE) pathway as one of the main factors affecting the impact strength of the disease [13]. Oxidative stress that occurs under previously mentioned tick-borne diseases induces intensified lipid metabolism, which significantly affects the lipidomic profile of patients [14,15]. Depending on individual properties, the production of lipid mediators is increased to a varying degree, which leads to the spread of signaling molecules throughout the body, inducing a mainly inflammatory reaction [16]. To describe the overall view of the changes induced by the pathogens transmitted by ticks, large-scale omics analyses are increasingly being used, which mainly concern metabolomic/lipidomic profiling [17,18,19]. However, proteins can also play a significant role in the development of many diseases. So far, several important pathways of protein signaling that are modified during TBE, Lyme disease, or anaplasmosis development have already been identified [19,20,21]. However, these results were based on changes in individual cell types infected by pathogens, and there is no overview of what happens in the human organism at all. Moreover, the mentioned pathogens can independently exist side-by-side in one vector, leading to infection and the development of more than one disease in one person. The described co-infections have frequently been noticed in domestic animals, with disastrous effects on health and life [22,23], but they are also being increasingly recognized in humans [24,25].

According to the above, the aim of this study was to evaluate the changes in the proteomic profile of human plasma induced by the development of tick-borne encephalitis (TBE) and to compare it with changes in the plasma proteome of TBE patients co-infected with other tick-borne pathogens. Due to the huge scope of proteomic research, this study concentrated on profiling the proteins and the analysis of protein structure modification connected with oxidative stress, including lipid peroxidation products binding and glutathionylation, induced by the development of the mentioned diseases. The obtained results may contribute to a better understanding of the changes in patients’ bodies, as well as to the fast identification of these diseases and effective therapy.

## 2. Results

### 2.1. Changes in Plasma Proteome of TBE and Co-Infected Patients

**Figure 1:**
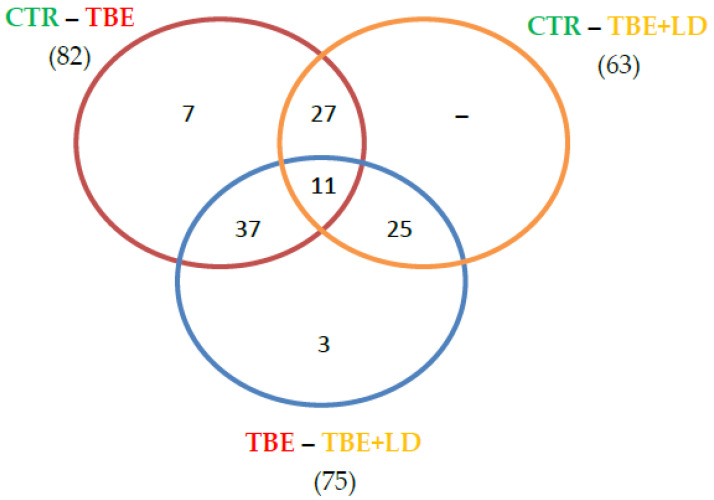
The distribution of the significantly changed proteins between each pair of the analyzed groups: control (CTR), patients with tick-borne encephalitis (TBE), and patients with TBE and co-infection (TBE+LD).

The obtained proteomic results allowed the identification and quantification of 492 proteins expressed in the analyzed plasma (Supplementary File S1). Within the quantified proteins, a total of 31 (0.2%) missing values were detected, and these missing values were replaced by 1/5 of the minimal positive values of their corresponding variables. Using ANOVA, among all quantified proteins, 110 were identified as statistically significantly different between the control (CTR), patients with tick-borne encephalitis (TBE), and patients with TBE and co-infection (TBE+LD). The distribution of the proteins in which expression was significantly changed between each of the two groups are shown in a Venn diagram (Figure 1). Out of the 82 proteins that differed between the TBE and CTR groups, 38 also differed between TBE+LD and CTR. The expression of only three proteins was specific to the TBE-TBE+LD comparison. Moreover, similar differences in the plasma proteomic profile of TBE and TBE+LD were identified based on volcano plots, where TBE was distinguished from CTR by 76 proteins, TBE+LD from CTR by 16 proteins, and TBE+LD from TBE by 46 proteins (Supplementary File S2). The list of these proteins with *p*-value and fold change is included in the supplementary files (Supplementary File S3).

**Figure 2:**
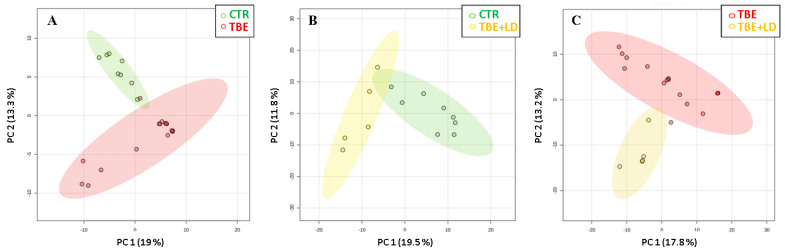
Principal component analysis (PCA) of plasma samples of patients with tick-borne encephalitis (TBE, n = 16) and patients with TBE and co-infection (TBE+LD, n = 5), as well as healthy donors constituting the control group (CTR, n = 8). The charts show the grouping of samples when analyzed CTR with TBE (**A**); CTR with TBE+LD (**B**), and TBE with TBE+LD (**C**).

**Figure 3:**
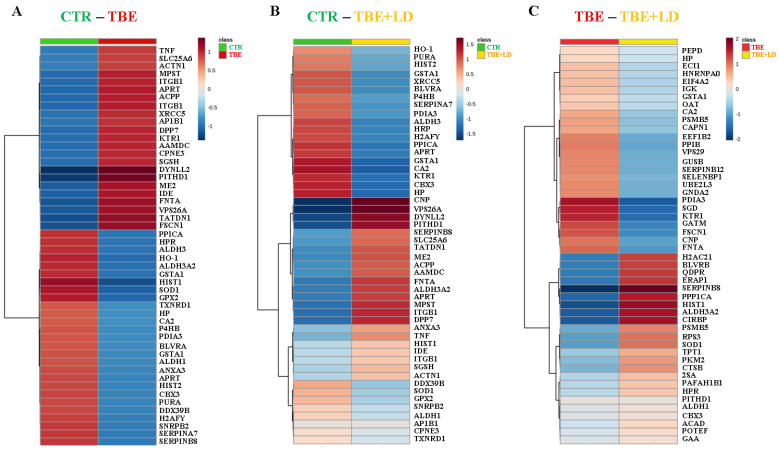
Heatmaps of top 50 proteins identified by ANOVA expressed in the plasma samples of the control group (CTR, n = 8), patients with tick-borne encephalitis (TBE, n = 16), and patients with TBE and co-infection (TBE+LD, n = 5). Heatmaps show the results when analyzed CTR with TBE (**A**); CTR with TBE+LD (**B**), and TBE with TBE+LD (**C**). Data showed the average protein intensity. The heatmaps with the intensity of proteins of the individual samples are included in the supplementary files (Supplementary File S4).

**Figure 4:**
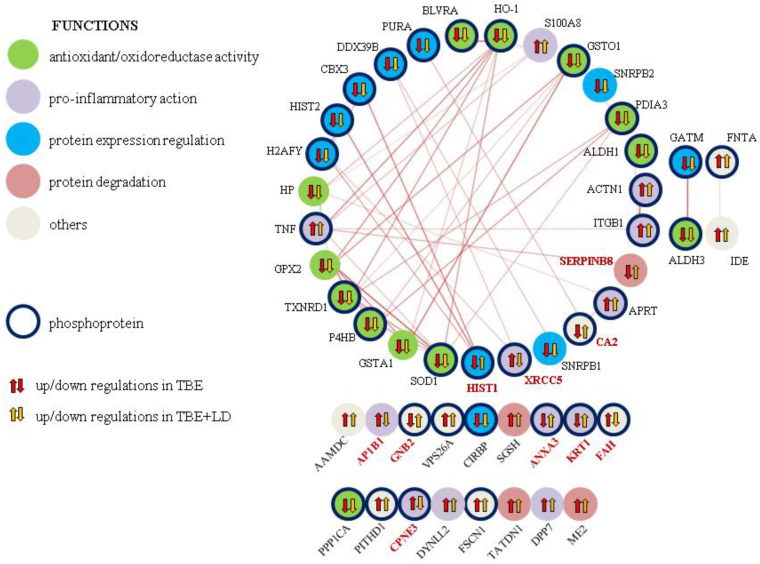
The protein network and biological functions of top proteins significantly changed in the plasma of patients with tick-borne encephalitis (TBE) and patients with TBE and co-infection (TBE+LD) compared to the healthy donors constituting the control group (CTR). The red marks the proteins whose expression changed differently in TBE and TBE+LD.

The mentioned differences in the proteins’ expression led to a clean separation of the observed study groups as a result of the principal component analysis (PCA) (Figure 2). To visualize the data and to retain the most contrasting patterns, heatmaps of the top 50 proteins ranked by *t*-tests were prepared (Figure 3). The biological functions of the proteins indicated in heatmaps, as well as the main pathway that they are involved in, are shown in Figure 4. The obtained results showed that the most strongly upregulated proteins in the plasma of TBE and TBE+LD patients were involved in the pro-inflammatory response and protein degradation, while the antioxidant proteins and factors responsible for protein biosynthesis were mainly downregulated. The main proteins whose expression differed between TBE and TBE+LD were: AP-1 complex subunit β-1 (AP1B1), calcium-dependent phospholipid-binding protein 3 (CPNE3), X-ray repair cross-complementing protein 5 (XRCC5), and fumarylacetoacetase (FAH)—all of which were upregulated in TBE and downregulated in TBE+LD; and serpin B8, histone H4 (HIST1), keratin (KRT1), annexin (ANXA3), guanine nucleotide-binding protein G(I)/G(S) (GNB2), and carbonic anhydrase 2 (CA2)—in which the changes in expression in TBE and TBE+LD were respectively reversed. Moreover, the greatest number of the indicated modified proteins were phosphoproteins, which accounted for 68% of these proteins (Figure 4).

**Figure 5:**
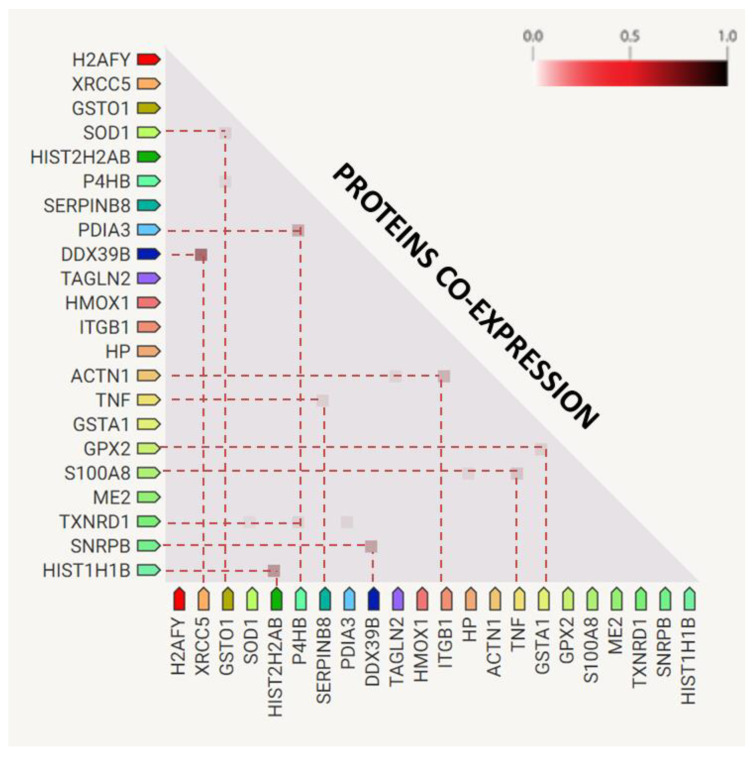
Co-expression of top significantly modified proteins identified in the plasma samples of the control group (CTR), patients with tick-borne encephalitis (TBE), and patients with TBE and co-infection (TBE+LD). The separate lines are used to mark proteins with the highest co-expression (>0.5).

The expression of modified proteins in the plasma of TBE or TBE+LD were partially correlated with each other (Figure 5). The following groups of proteins had the strongest correlation rate (>0.5): small nuclear ribonucleoprotein (SNRPB)-XRCC5–spliceosome RNA helicase (DDX39B); HIST1–HIST2; serpin B8-S100A8–tumor necrosis factor α (TNFα); actinin-1 (ACTN1)–integrin β1 (ITGB1); protein disulfide-isomerase 4 (P4HB)–protein disulfide-isomerase A3 (PDIA3)–thioredoxin reductase 1 (TXNRD1); glutathione S-transferase A1 (GSTA1)–glutathione peroxidase-like peroxiredoxin 2 (GPX2).

### 2.2. Protein Adducts Formation as an Effect of TBE and TBE+LD Infections

**Figure 6:**
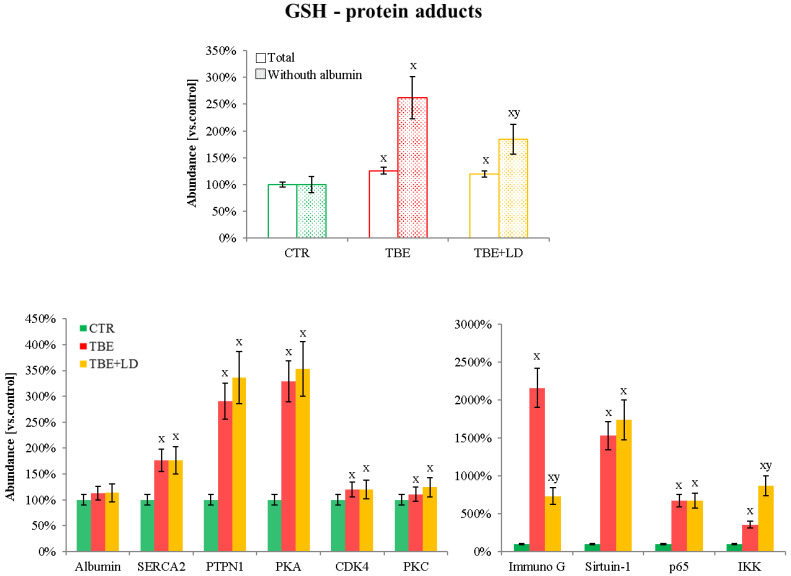
The level of protein modification by glutathione (GSH) in the plasma samples of the control group (CTR, n = 8), patients with tick-borne encephalitis (TBE, n = 16), and patients with TBE and co-infection (TBE+LD, n = 5). Mean values ± SD are presented. ^x^ statistically significant differences vs. CTR, *p* < 0.05; ^y^ statistically significant differences vs. TBE, *p* < 0.05.

**Figure 7:**
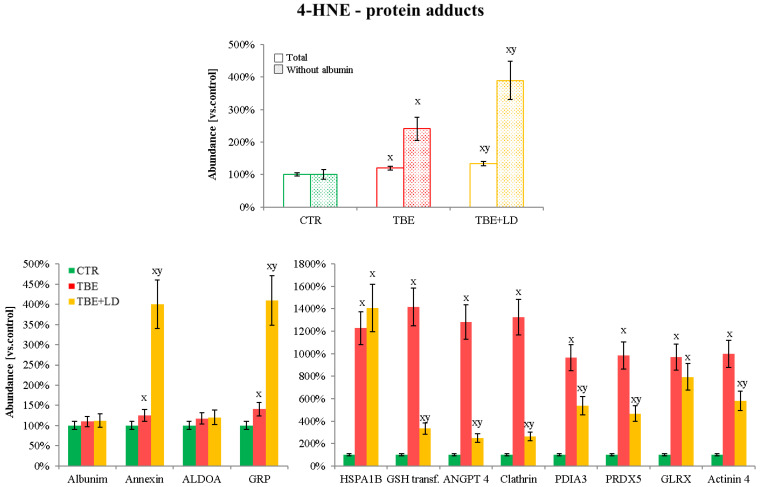
The level of protein modification by 4-hydroxynonenal (4-HNE) in the plasma samples of the control group (CTR, n = 8), patients with tick-borne encephalitis (TBE, n = 16), and patients with TBE and co-infection (TBE+LD, n = 5). Mean values ± SD are presented. ^x^ statistically significant differences vs. CTR, *p* < 0.05; ^y^ statistically significant differences vs. TBE, *p* < 0.05.

The obtained results showed that TBE development in patients from whom analyzed plasma samples were collected was accompanied by disturbances in redox balance. Regardless of the decrease in antioxidant proteins, an enhanced level of pro-oxidative proteins, mainly as a result of GSH or 4-HNE binding, was also observed (Figure 6 and Figure 7). In both cases, the main modified protein was albumin, whose adducts accounted for approximately 90% of all modifications. However, other proteins were also bound to GSH or 4-HNE. It was found that TBE development caused a 2.5 times increase in the total (excluding albumin) level of GSH–protein adducts, while TBE+LD increased this parameter by 1.8 times (Figure 7). The proteins most modified by GSH were: sarco/endoplasmic reticulum Ca^2+^-ATPase (SERCA2), tyrosine-protein phosphatase non-receptor type 1 (PTPN1), protein kinase A (PKA), protein kinase C (PKC), cyclin-dependent kinase 4 (CDK4), sirtuin-1, p65, inhibitor of nuclear factor kappa-B kinase subunit beta (IKK), and immunoglobulin superfamily member 1 (Immuno G). The modification of these proteins was increased in both TBE and TBE+LD patients at a similar level, excluding GSH adducts with IKK, which were formed in a smaller degree in TBE than in TBE+LD, and GSH adducts with Immuno G, which were formed in a bigger degree in TBE compared to TBE+LD (Figure 6).

Moreover, the formation of protein adducts with 4-HNE was also enhanced in the plasma of TBE/TBE+LD patients, in comparison to the control group. TBE development increased the total level of 4-HNE–protein adducts 2.5 times, and TBE+LD around 4 times (Figure 7). Among the proteins much more strongly modified by 4-HNE in TBE+LD patients than in TBE were annexin A1 and 78 kDa glucose-regulated protein (GRP). However, in the plasma of TBE patients, 4-HNE–protein adducts were created more significantly on proteins such as: glutathione S-transferase (GSH transferase), angiopoietin-4 (ANGPT4), clathrin, PDIA3, actinin-4, and peroxiredoxin-5 (PRDX5). Only the 4-HNE-heat shock 70 kDa protein 1B (HSPA1B) and 4-HNE–glutaredoxin (GLRX) adducts were increased by TBE and TBE+LD development at the same level (Figure 7).

## 3. Discussion

All tick-borne diseases, both bacterial and viral, pose a huge threat to the health and life of infected patients. Their first symptoms are rarely unambiguous and appear with a delay in relation to a tick bite. Moreover, serological tests have some limitations and are not perfect diagnostic tools. Therefore, there is still a search for precise descriptions of the development mechanism of tick-borne diseases, which would enable the development of fast and accurate diagnostic tools. Thanks to comprehensive proteomic research, we are able to describe the changes taking place in the plasma proteome of TBE patients and those that significantly differentiate patients with TBE and patients co-infected with tick-borne bacterial pathogens (TBE+LD), even despite the small number of tested co-infected samples.

### 3.1. Changes in Plasma Proteome of TBE and Co-Infected Patients

#### 3.1.1. Molecules Involved in Protein Expression Regulation

The obtained results indicate that around 20% of the top modified proteins in the plasma of TBE-infected patients are involved in protein expression regulation. In the vast majority of these proteins, a reduction in their level, regardless of mono-infection (TBE) or co-infection (TBE+LD), compared to the plasma of the control group is observed. This also applies to the proteins responsible for chromatin conformation, such as chromobox protein 3 (CBX3) and histones (HIST2, H2AFY). The decreased level of their expression disrupts the transcriptional processes in the cells of infected patients. Moreover, in the case of other tick-borne parasites, the dynamic methylation of histones has been indicated [26]. The combination of a decreased level of histones and their methylation shows that tick-borne infections lead to the impairment of gene expression in the patient’s organism, especially those responsible for apoptotic processes in the blood cells, which facilitates further spreading of the virus in the body and favors disease development [27]. On the other hand, at the same time, TBE and TBE+LD lead to a decrease in the level of cold-inducible RNA-binding protein (CIRBP), which is involved in pro-apoptotic signaling [28]; thus, the described effect is additionally deepened. The gene’s expression can be affected by the development of TBE and TBE+LD not only at the transcription level, but also at the level of the molecules involved in the regulation of the translation and post-translation stages. This is visible in the case of the decreased level of spliceosome RNA helicase (DDX39B) and the nuclear ribonucleoproteins (SNRPB1/2) involved in pre-mRNA splicing as a component of the spliceosome. However, so far, the importance of spliceosome-building proteins in the development of tick-borne diseases has been defined only in terms of their heterogeneity and their allowing viruses or bacteria to better colonize the organism [29,30].

#### 3.1.2. Expression of Proteins Involved in Antioxidant Capacity

Plasma proteome profiling reveals a number of proteins whose expression is altered during the development of TBE, as well as TBE with bacterial co-infections. This group includes many proteins involved in the maintenance of redox homeostasis, mainly antioxidant enzymes (superoxide dismutase (SOD), TXNRD1, GPX2, GSTA1), the expression of which is strongly reduced in both patients with TBE and those with co-infections. This is consistent with data in the literature that shows a strong increase in oxidative stress accompanying TBE and TBE+LD development, observed in the basic markers of oxidative stress, including an increase in reactive oxygen species (ROS) generation and a decrease in the reduced form of glutathione and thioredoxin, as well as the effectiveness of their antioxidant systems [31]. On the other hand, only a reduced SOD activity has so far been observed in HGA patients [32]. Despite the lack of other data in the literature regarding the influence of tick-borne infections on the antioxidant capacity of the blood plasma of patients, the observed increase in ROS-dependent lipid peroxidation (estimated as 4-HNE, malondialdehyde) in TBE and LD [16,33,34] indicates the presence of oxidative stress in the bodies of patients as a consequence of redox equilibrium shifting towards oxidation processes.

In all these diseases, it seems particularly dangerous when both thioredoxin-dependent and glutathione-dependent antioxidant systems are downregulated at the same time. Under physiological conditions, both of these systems work together to eliminate ROS and prevent oxidative stress and, consequently, the participation of ROS in oxidative modifications of the membrane phospholipids, thus reducing the destructive effect of a potentially developing disease. At the same time, the failure of one of these systems is eliminated by the action of the other [35]; however, the simultaneous impairment of both may even lead to neurodegeneration [36]. Moreover, the activation of both these systems in cells cultured in vitro contributes to the protection of phospholipids against oxidation, fragmentations, and lipid mediator generation [37]. In addition to the classic antioxidant role of these systems, some thioredoxin- and glutathione-dependent enzymes are also able to control post-translational modifications, including phosphorylation [38]. Whereas the results obtained in our study show that tick-borne diseases decrease the level of antioxidant proteins, their activity is strongly connected with their phosphorylation. This is especially important in the case of heme oxygenase (HO-1), which, on one side, is activated by phosphorylation and whose level, on the other, is strongly correlated with phospho-Nrf2 transcriptional activity. The activation of the Nrf2/HO-1 pathway following a tick bite is the initial reaction of skin cells to tick saliva [39]; however, as the obtained results show, both viral and bacterial infections cause a significant decrease in the HO-1 plasma level, resulting in oxidative stress.

#### 3.1.3. Expression of Proteins Responsible for Inflammation

According to the oxidative stress that occurs in patients infected with TBE and other tick-borne diseases, an uncontrolled intensification of lipid oxidative metabolism that results in an increase in lipid mediators, such as neuroprostanes, is observed [16,33,34]. This is accompanied by a significant increase in the level of pro-inflammatory factors, such as NFκB and TNFα [31]. In the present study, it is shown that in TBE as well as co-infected patients, protein pro-inflammatory mediators are also enhanced. This concerns, e.g., KRT1, TNFα, S100A8, ANXA3, and ACTN1. It is known that KRT1 induces inflammation and innate immunity in the skin [40], especially following oxidative stress induced by tick bite. Additionally, as a result of a TNFα increase in the plasma, the signal that induces pro-inflammatory reactions in the cells is distributed throughout the organism. Moreover, TNFα, after attaching to the appropriate receptors, stimulates NFκB activity, leading to the biosynthesis of pro-inflammatory cytokines, including TNFα. This results in a reverse intensification of pro-inflammatory signaling [41]. TNFα is also co-expressed with another pro-inflammatory protein, S100A8 [42]. This molecule can function as a cytokine that induces the growth, proliferation, and activation of cells involved in the immune response, such as lymphocytes. The parallel upregulation of ANXA3 in TBE/TBE+LD patients carries an increased risk of growth of the neutrophil–lymphocyte ratio, infiltrating all body tissues [43], the migration of which is regulated by actin-binding ACTN1, whose expression is also increased in TBE/TBE+LD plasma patients. Upregulated ACTN1 level also stimulates the action of integrin β1, which are expressed specifically on leukocytes during inflammation and promote cellular adherence, phagocytosis, and cytotoxic effects, especially during bacterial infections [44].

#### 3.1.4. Proteins Differentiating TBE from Co-Infected Patients

Despite all of the above-mentioned changes in the proteomic profile of the plasma of TBE/TBE+LD-infected patients, there are also proteins that significantly differentiate the profiles of patients with only TBE, as well as TBE and bacteria co-infected patients. One of these proteins is protease inhibitor-serpin (SERPINB8), whose enhanced expression accompanies tick-saliva injection [45]; but after the tick bite, its expression is induced mostly in bacterial infections [46] and not in viral, due to the fact that its pro-survival action limits the spread of viruses [47,48]. A similar situation is observed in the case of GNB2, which is involved as a modulator or transducer in various transmembrane signaling systems, including inflammasome creation, especially in bacterial infections but not in viral [49]. Moreover, viral TBE infection decreases CA2, which catalyzes the conversion of carbon dioxide and bicarbonate to maintain an acid–base balance in the blood and other tissues. As a result of an acid–base imbalance, the rupturing of cell membranes ensures effective virulence, which is detrimental for cellular parasites, whose co-infection significantly increases CA2 expression, as observed in this study and in data in the literature [50,51,52].

On the other hand, TBE infection significantly increases the level of AP1B1 and CPNE3 proteins, which are reduced in co-infected patients. AP1B1 plays a role in protein sorting following maturation and endo-/exosomes creation [53], while CPNE3 is responsible for cell migration in response to growth factor stimulation [54]. As a result of the increased expression of both proteins, viruses can spread throughout the body with greater efficiency. However, co-infection with bacteria significantly reduces the level of these proteins, which may restrict the migration of immune cells. Additionally, similar results are observed for the protein responsible for amino acid degradation, FAH, whose increased expression following viral infection promotes TBE vector virulence [55]; however, there are no data in the literature to suggest why, in the case of bacterial infections/co-infections transmitted by ticks, there is such a reduced level of FAH. Putting these facts together, a search among the discussed proteins for potential biomarkers that differentiate TBE from co-infected patients can be suggested.

### 3.2. Effect of GSH and 4-HNE Action on Protein Adducts Formation

Disturbances in the expression of antioxidant proteins observed in the course of tick-borne viral and bacterial infections, including TBE or LD, may also be accompanied by a decrease in the activity of antioxidant enzymes, including glutathione peroxidase (GSH-Px) [16,33,34]. GSH-Px counteracts oxidative modifications of phospholipids; therefore, its deficiency is usually accompanied by a reduced level of polyunsaturated fatty acids and an increased level of reactive aldehydes, including 4-HNE, which can interact with the nucleophilic elements of proteins with the formation of adducts [16,33,34]. The decreased activity of GSH-Px may be the result of GSH deficiency, which is a co-factor of this enzyme, but also indirectly participates in metabolism, including glutathionylation [56]. As a result, proteins with biologically important functions can be modified by attaching GSH or 4-HNE molecules to their structure.

#### 3.2.1. GSH–Protein Adducts in Plasma of Patients Infected with Tick-Borne Diseases

The increased formation of GSH–protein adducts in the plasma of patients with TBE, as well as those with co-infections, observed in this study may be based on the same mechanisms of the proteins’ glutathionylation that is observed in viruses and is inseparable from their virulence [57]. Similar results, but on a smaller scale, are observed for some bacteria [57], which in the case of *B. burgdorferi* also induces protein glutathionylation in the host, leading to cytokine overproduction and inflammation [58]. Glutathionylation can result in enzyme inhibition because it often concentrates on the cysteine residues localized in their active centers [59]. This is of the greatest importance for pro-inflammatory signaling based on the nuclear factor κB (NFκB) pathway [60]. In this study, an increase in the level of GSH adducts with NFκB and its inhibitors (GSH-p65 and GSH-IKK) was demonstrated in the plasma of TBE patients. Glutathionylation of these proteins induces the activation of the NFκB pathway, resulting in an exacerbation of systemic inflammation, which is much more potent in co-infected patients, where GSH modifies IKK at a higher level than in TBE-only patients. Additionally, in the plasma of patients infected with the examined tick-borne diseases, glutathionylated kinases, including PKA, PKC, and CDK4, are found in high levels. This results in disturbances in the phosphorylation-dependent signaling pathways. So far, the glutathionylation of many various kinases has been described as a factor leading to loss of their enzymatic activity [61,62,63]. In the case of PKA and PKC, glutathionylation of cysteine 199 in the activation loop of the catalytic subunit blocks the phosphotransfer reaction and inhibits the creation of an intramolecular disulfide bond between cysteines 199 and 343 [64]. As a result of PKA and PKC negative regulation, the PI3K and protein kinase B (Akt) signaling pathways are activated [65], which in some cases can even result in cancer development [66]. Moreover, through PI3K/Akt activation, TBE virus replication and transmission are increased [67], regardless of bacterial co-infection. Additionally, the created GSH-CDK4 adducts influence the conformation of the ATP binding site in this kinase and also inhibit CDK4 activity, thus spreading information about a disturbance in the cell cycle throughout the organism [64]. On the other hand, the glutathionylation of disulfide bonds in plasma immunoglobulins (GSH-immunoG), as one of the markers of redox disturbance in the organism [68], shows a significantly stronger increase in TBE patients than in co-infected patients. GSH-immunoG adducts from plasma are translocated into cellular endoplasmic reticulum (ER), where they stimulate activating transcription factor 6 (ATF6), resulting mainly in the chaperone’s transcription [69]. This induces in TBE patients a stronger self-protective response than in the case of viral–bacterial co-infections.

#### 3.2.2. 4-HNE–Protein Adducts in Plasma of Patients Infected with Tick-Borne Diseases

Described before, the enhanced level of the electrophilic and highly reactive lipid peroxidation product, 4-HNE, favors binding this molecule to proteins involved in pathways that are essential for the functioning of individual cells, as well as whole organisms, including pro-inflammatory and pro-apoptotic signaling [70]. The results presented in this study show that TBE+LD infection causes an increase in the formation of 4-HNE–protein adducts, which in the case of bacterial co-infections (TBE+LD) is almost doubled. This is possible mainly due to the large number of adducts formed on annexin A1 and GRP. Annexin A1 plays an important role in the regulation of the inflammatory process, based on inflammation blanking due to its inhibitory action on phospholipase A2 (PLA2) and decreasing the level of lipid pro-inflammatory factors [71]. Therefore, annexin A1 modifications by 4-HNE may interfere with its anti-inflammatory properties. Moreover, other studies show that 4-HNE favors protein phosphorylation [72], which in the case of annexin A1 often cause its inactivation [71]. Previously, in the plasma of patients infected with NB, the activities of the annexin-dependent enzyme, PLA2, have been found to be increased [34], which has not been observed in the plasma of TBE patients [16]. This suggests that the formation of 4-HNE–annexin A1 adducts, induced by viral and bacterial infection, induces an inflammatory response in the body through the PLA2-mediated pathway, as opposed to TBE. On the other hand, 4-HNE in the plasma of co-infected patients also significantly strongly binds to GRP, which, despite of its dependence on glucose levels, is also an immunoglobulin protein with a chaperone activity and plays a role in the retrograde transport across the membrane of aberrant proteins destined for degradation by the proteasome. GRP synthesis is induced mainly under stress conditions, which leads to the accumulation of damaged polypeptides [73]. An increased 4-HNE level mediates unfolded or destructed protein expression, leading to the overexpression of GRP in both viral [74,75] and bacterial infections, including from *Borrelia* spp. [76]. However, previously described 4-HNE–GRP adducts influence the activity of GRP via molecular aggregation [77], which, due to the obtained results in the case of tick-borne disease, has the most significant effect only for co-infected patients, where the body’s GRP-dependent protective response is most strongly inhibited.

4-HNE, generated during tick-borne diseases, also binds to other metabolically important proteins, including GSH transferase, ANGPT4, clathrin, PDIA3, actinin-4, and PRDX5. In all mentioned cases, the 4-HNE–protein adducts level is higher in TBE than in co-infected patients, which certainly results from the specificity of the selected proteins. Most of these proteins are indirectly involved in antioxidant response, such as GSH transferase, PDIA3, or PRDX5, and the 4-HNE, as a pro-oxidative signaling molecule, depending on the concentration, usually induces these enzymes’ activity [70]. In TBE/TBE+LD diseases, 4-HNE also modifies structural proteins, which can indirectly affect the functioning of the antioxidant system, such as actinin-4, which works through activation, e.g., peroxisome proliferator-activated receptor γ (PPARγ). As a result of 4-HNE binding, actinin-4 loses its activity and negatively affects signaling and intercellular cell condition [78]. These modifications are observed in TBE at a higher level than in the case of plasma samples from co-infected patients, which suggests that viral–bacterial co-infection induces in the body such a strong reaction and exhaustion of its defense mechanisms that even 4-HNE-dependent signaling is not enough to activate the antioxidant system. Moreover, the complex induction of the immune system against viral and bacterial antigens can trick the antioxidant system into receiving conflicting signals; however, the study group of the co-infected patients, as well as the proteomic approach, were not extensive enough to identify the precise mechanism, and this was a limitation of our study, so further analyses are required.

## 4. Materials and Methods

### 4.1. Samples Collection

Blood samples were collected from a group of 21 patients (10 female and 11 male) with a mean age of 42 years (range 22–63), treated in years 2019–2021 by the Department of Infectious Diseases and Neuroinfections, Medical University of Bialystok, Poland. Patients were divided into two groups: TBE and TBE co-infected with other tick-borne pathogens, including *B. burgdorferi* (LD, NB) and *A. phagocytophilum* (HGA). TBE was diagnosed according to European Academy of Neurology (EAN) guidelines [79], based on clinical symptoms, positive serology, and lymphocytic pleocytosis in the cerebrospinal fluid (CSF). LD and NB were defined on the basis of clinical presentation of erythema migrans, fulfilled criteria for neuroborreliosis, or the presence of anti-*Borrelia burgdorferi* antibodies after a tick bite [80,81]. HGA was diagnosed according to the case definition by the CDC when all three criteria were fulfilled (https://wwwn.cdc.gov/nndss/conditions/ehrlichiosis-and-anaplasmosis/case-definition, accessed on 2 September 2021):

1. Clinical presentation: A tick-borne illness characterized by acute onset of fever and one or more of the following symptoms or signs: headache, myalgia, malaise, anemia, leukopenia, thrombocytopenia, or elevated hepatic transaminases.
2. Exposure: History of having been in potential tick habitat in the 14 days prior to the onset of illness or history of tick bite.
3. Laboratory criteria for diagnosis: Detection of *A. phagocytophilum* DNA in a clinical specimen via amplification of a specific target by polymerase chain reaction (PCR) assay (a nested PCR directed to a 546 bp fragment of the *16S rRNA* gene of *A. phagocytophilum* was performed (Blirt-DNA Gdańsk, Poland) in a SensoQuest LabCycler (SensoQuest, Göttingen, Germany)).

Co-infection was diagnosed if one patient was infected with at least two different pathogens. To create a control group, blood was also collected from group of 8 healthy donors that were age- and gender-matched to the study group. The demographic and clinical characteristics of the patients and the control group, as well as a comparison of their laboratory data, are presented in Table 1 and Table 2.

The study was conducted in accordance with the Declaration of Helsinki and was approved by the Local Bioethics Committee, Medical University of Bialystok (Bialystok, Poland), No. R-I-002/169/2018. Written informed consent was obtained from all the patients and healthy donors.

Blood samples were collected into ethylenediaminetetraacetic acid (EDTA) tubes and centrifuged at 3000× *g* (4 °C) to obtain the plasma. Albumin was separated from the samples using ProteoExtract^®^ Albumin Removal Kit (Calbiochem, Darmstadt, Germany) according to the manufacturer’s protocol. The total protein concentration of each sample devoid of albumin or separated albumin was measured by Bradford assay [82], and the amounts containing 50 µg proteins were used for proteomic analysis, while samples containing 100 µg proteins were used for glutathione (GSH) adducts immunoprecipitation.

### 4.2. Isolation of GSH–Protein Adducts

Samples were pre-cleaned with protein A-agarose to remove molecules that could nonspecifically react with the substance. Protein A-agarose was removed by 1 min centrifugation (10,000× *g*, 4 °C). Next, the primary antibody against GSH (1:1000; Sigma-Aldrich; St. Louis, MO, USA) was added, and the samples were incubated for 1h at 4 °C. To precipitate the proteins bound with antibodies, protein A-agarose was added and incubated overnight. The next day, the samples were centrifuged (10 min, 10,000× *g*, 4 °C), and the obtained pellet was received as proteins immunoprecipitated with GSH.

### 4.3. Protein Digestion and Proteomic Analysis

Before digestion, the proteins contained in all types of samples were denatured by mixing with 8 M urea. Next, the proteins were reduced with 10 mM 1,4-dithiothreitol (DTT) and alkylated by incubation with 50 mM iodoacetamide (IAA). To stop the alkylation, the DTT was again added. Following fourfold dilution, the samples were in-solution digested overnight (37 °C) with trypsin (Promega, Madison, WI, USA) in a ratio of 1:50 (trypsin: proteins). To stop the trypsinization, 10% formic acid (FA) was added in an amount ensuring a final concentration in the samples of 0.1% [83]. The obtained peptide mixture was dried under inert gas and dissolved in 5% acetonitrile (ACN) with 0.1% FA.

The peptides were separated using a high-performance liquid chromatography system (Ultimate 3000; Dionex, Idstein, Germany) on μPAC 200 column (PharmaFluidics, Ghent, Belgium) at a flow rate of 0.300 µL/min with 200 cm long separation channel and 5 µm silicon pillars. The solvents gradient started at 3 min and increased to 60% eluent B (90% ACN + 0.03% FA) for 90 min. Eluent A contained 5% ACN with 0.1% FA. Eluted peptides were analyzed using a Q Exactive HF mass spectrometer with an electrospray ionization source (ESI) (Thermo Fisher Scientific, Bremen, Germany), and the conditions of the analysis for peptide identification have previously been described in detail [84].

### 4.4. Protein Identification and Label-Free Quantification

Raw data were acquired with the Xcalibur software (Thermo Fisher Scientific, Bremen, Germany) and analyzed using Proteome Discoverer 2.0 (Thermo Fisher Scientific, Seattle, WA, USA). Input data were searched against the UniProtKB-SwissProt database (taxonomy: Homo sapiens, release 2021-02). The following search parameters were used for the identification of proteins: peptide mass tolerance set to 10 ppm; MS/MS mass tolerance set to 0.02 Da; up to two missed cleavages allowed; a minimal peptide length set to six amino acids; the minimal number of identified unique peptides for each protein set to two peptides; and cysteine carbamidomethylation and carboxymethylation, methionine oxidation, as well as 4-hydroxynonenal (4-HNE)–cysteine/lysine/histidine set as dynamic modifications [85,86]. Protein label-free quantification was performed according to the signal intensities of the precursor ions. The levels of 4-HNE–protein adducts were estimated based on the peak intensity of peptides modified by 4-HNE identified in at least 80% of the samples in each group.

### 4.5. Statistical Analysis

**Figure 8:**
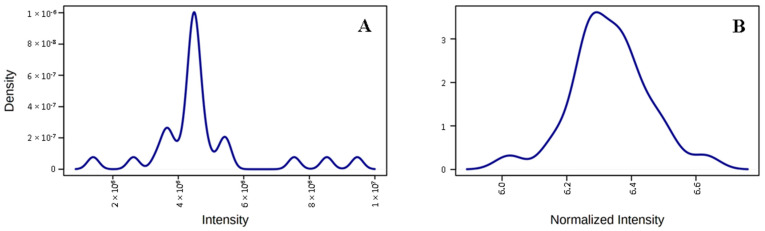
The distribution of the density of the plasma samples of patients with tick-borne diseases (total n = 21) and of healthy donors (n = 8) before (**A**) and after log-transformation, auto-scaling, and normalization by the sum (**B**).

The results from individual protein label-free quantification using the open-source software MetaboAnalyst 5.0 (http://www.metaboanalyst.ca, accessed on 20 December 2021) [87] were log-transformed, auto-scaled (mean-centered and divided by the standard deviation of each variable), and normalized by the sum of the proteins’ intensities obtained for each sample, which ensured the samples’ normal distribution (Figure 8; Supplementary File S5). The same software was used for biostatistical analysis, including *t*-test/univariate analysis one-way (ANOVA (*p* < 0.05)), Fisher’s least-significant differences (LSD), the false discovery rate (FDR) < 5%, principal component analysis (PCA), heatmaps, and volcano plots creation. Protein functions, co-expression, and networks were made using the STRING database (STRING 11.0) [88] and Cytoscape (Cytoscape 3.8.2) [89].

## 5. Conclusions

The results obtained in this study showing changes in the plasma proteome of TBE-infected and bacterial co-infected patients largely elucidate the changes that occur in the human body during the development of these diseases. It is especially important due to the fact that patients presenting to a clinician who studies the metabolic changes associated with tick-borne diseases usually have some symptoms of the disease that can be alleviated by generally available agents, which makes it difficult to obtain reliable data. It is also visible in this study where the group of infected patients, especially with viral–bacterial co-infection, contains only single cases. Regardless of the size of the study group, the differences in the profiles between TBE and TBE+LD indicate that the changes that occur during the development of these diseases are divergent and require different treatment, which should be of particular interest in terms of the further search for innovative diagnostic tools for co-infection, as well as for potential vaccines. However, the complex nature of these diseases does not allow the indication of one specific protein biomarker of these diseases. The obtained results give hope that the analysis of a larger population group of TBE and bacterial co-infections, including a quantitative analysis, will allow the identification of the protein/proteins responding to various pathogens in a different manner. It should be particularly interesting from the point of view of the further search for innovative diagnostic tools for co-infections, as well as dedicated pharmacotherapy and finding potential vaccines.

## Figures and Tables

**Table 1:** Demographic and clinical characteristics of TBE and TBE co-infected (TBE+LD) patients compared to healthy subjects (CTR, control).

	CTR	TBE	TBE+LD
Age (years)	40.25 ± 7.42	42.75 ± 9.33	42.5 ± 15.24
Sex, female/male	4/8 female (50%) 4/8 male (50%)	7/16 female (43%) 9/16 male (57%)	3/5 female (60%) 2/5 male (40%)
Place of residence (urban/rural area)		7/16 urban (44%) 10/16 rural (56%)	3/5 urban (60%) 2/5 rural (40%)
Noticeable tick bite	0/8 (0%)	9/16 (56%)	5/5 (100%)
Time since tick bite (days)		25 ± 16.19	17.5 ± 4.95
Duration of hospitalization (days)		12.56 ± 2.13	12.6 ± 2.07
Duration of symptoms (days)		8.53 ± 8.08	4.33 ± 3.21
Clinical form			
Meningitis	0/8 (0%)	10/16 (62%)	4/5 (83%)
Meningoencephalitis	0/8 (0%)	6/16 (38%)	1/5 (17%)
Meningoencephalomyelitis	0/8 (0%)	0/16 (0%)	0/5 (0%)
Clinical presentation			
Headache	0/8 (0%)	16/16 (100%)	5/5 (100%)
Fever	0/8 (0%)	15/16 (94%)	4/5 (80%)
Neck stiffness	0/8 (0%)	13/16 (81%)	3/5 (60%)
Kernig’s sign	0/8 (0%)	5/16 (31%)	1/5 (20%)
Vertigo	0/8 (0%)	7/16 (44%)	0/5 (0%)
Nausea	0/8 (0%)	7/16 (44%)	2/5 (40%)
Vomiting	0/8 (0%)	6/16 (38%)	0/5 (0%)
Ataxia	0/8 (0%)	5/16 (31%)	0/5 (0%)
Tremor	0/8 (0%)	4/16 (25%)	0/5 (0%)
Hyperesthesia	0/8 (0%)	2/16 (13%)	1/5 (20%)
Muscle pain	0/8 (0%)	1/16 (6%)	0/5 (0%)
Joint pain	0/8 (0%)	1/16 (6%)	0/5 (0%)
Consciousness disturbances	0/8 (0%)	1/16 (6%)	0/5 (0%)
Skin lesion	0/8 (0%)	0/16 (0%)	1/5 (20%)
Muscle weakness	0/8 (0%)	0/16 (0%)	1/5 (20%)

**Table 2:** Comparison of laboratory data of TBE and TBE co-infected (TBE+LD) patients with healthy subjects (CTR).

	CTR	TBE	TBE+LD
Complete blood count			
WBC [10^3^/μL]	4.00–10.00	10.35 ± 2.27	7.41 ± 1.47
Neutrophils [%]	40.0–72.0	70.56 ± 10.21	61.2 ± 7.54
Lymphocytes [%]	18.00–48.00	18.8 ± 9.27	25.58 ± 4.77
Monocytes [%]	2.50–10.00	9.49 ± 2.68	10.86 ± 2.18
RBC [10^6^/μL]	4.00–5.50	4.29 ± 0.33	4.33 ± 0.61
HGB [g/dL]	12.00–16.00	12.91 ± 1.08	12.74 ± 1.39
PLT [10^3^/μL]	130–350	251 ± 49.17	280 ± 57.92
CRP [mg/L]	0.00–5.00	12.16 ± 18.48	1.56 ± 1.28
Glucose [mg/dL]	70–110	96 ± 10.47	92.67 ± 8.5
Creatinine [mg/dL]	0.50–0.90	0.89 ± 0.17	0.79 ± 0.09
ALT [U/I]	0–31	25 ± 27.3	17.75 ± 11.27
AST [U/I]	0–32	16.2 ± 7.01	17 ± 3.67

## Data Availability

The mass spectrometry proteomics data have been deposited to the ProteomeXchange Consortium via the PRIDE partner repository with the dataset identifier PXD028803.

## Supplementary Materials

The following supporting information can be downloaded at https://www.mdpi.com/article/10.3390/ijms23084374/s1.
