# Supplementary figures and images for: Plasma Proteomic Profile of Patients with Tick-Borne Encephalitis and Co-Infections
Source: Int J Mol Sci. 2022 Apr 15;23(8):4374. doi: 10.3390/ijms23084374 (PMC9031133; doi:10.3390/ijms23084374)

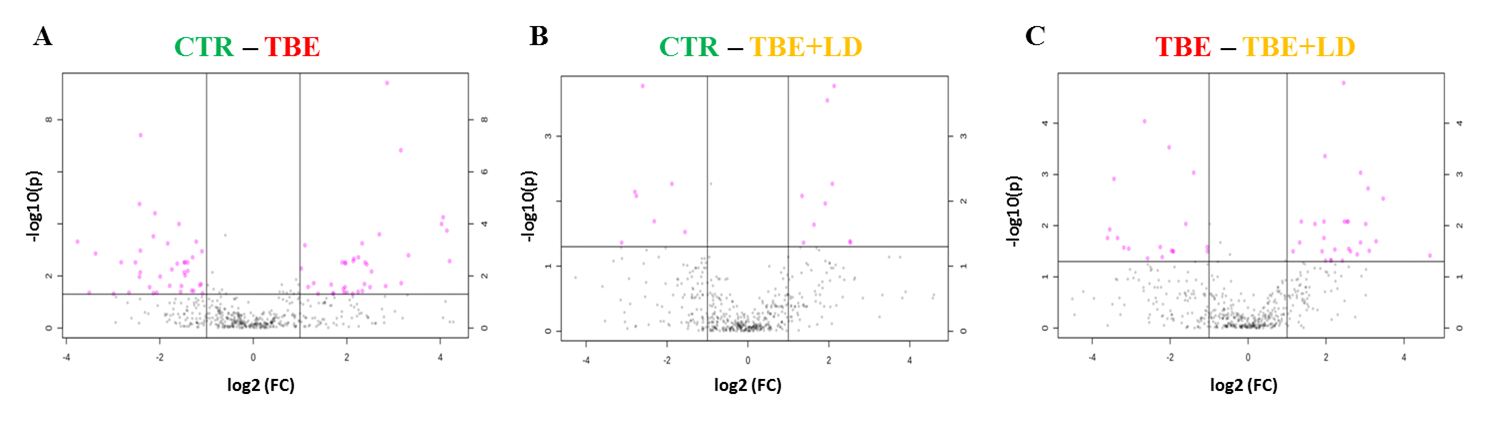

Supplement: Supplementary file 1 [file ijms-23-04374-s001.zip › S2.tif]

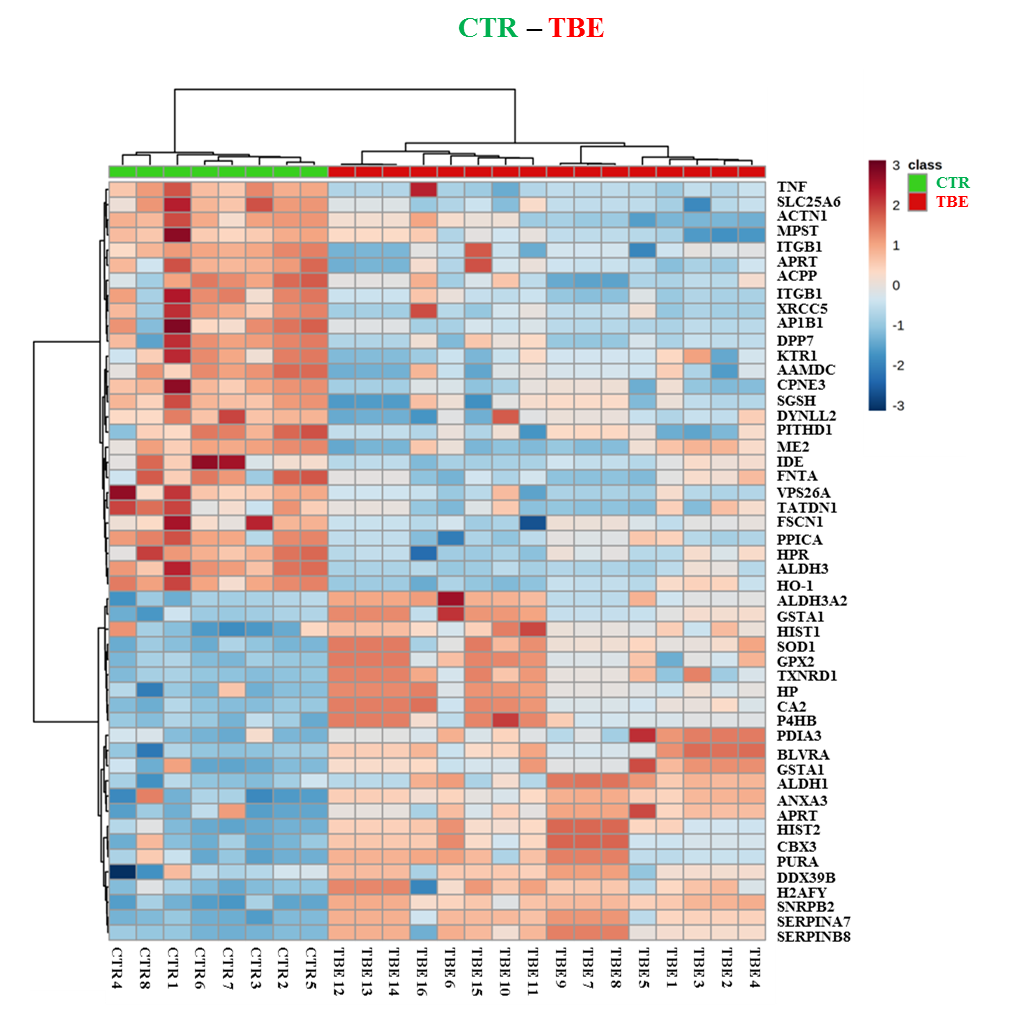

Supplement: Supplementary file 1 [file ijms-23-04374-s001.zip › S4 Fig A.tif]

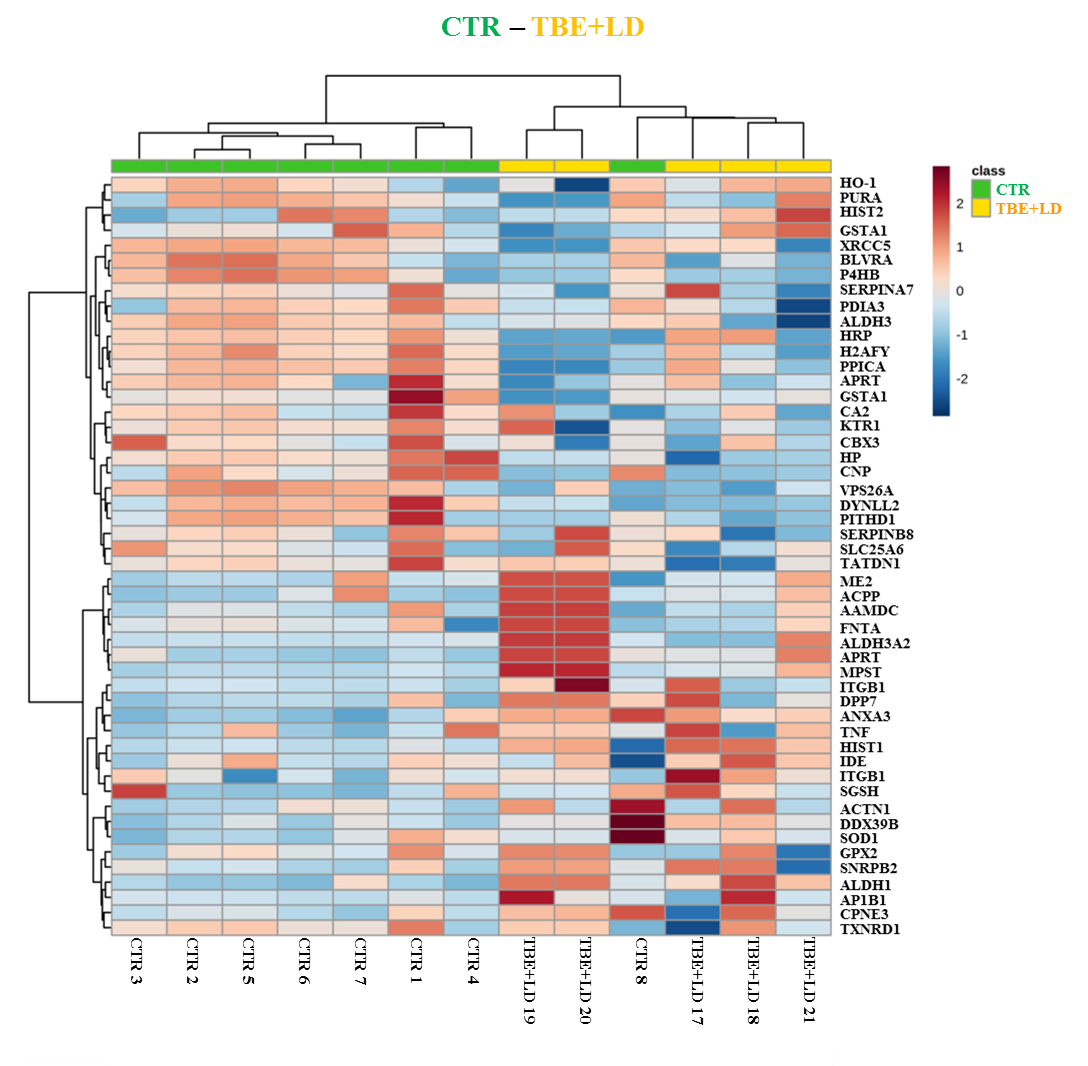

Supplement: Supplementary file 1 [file ijms-23-04374-s001.zip › S4 Fig B.tif]

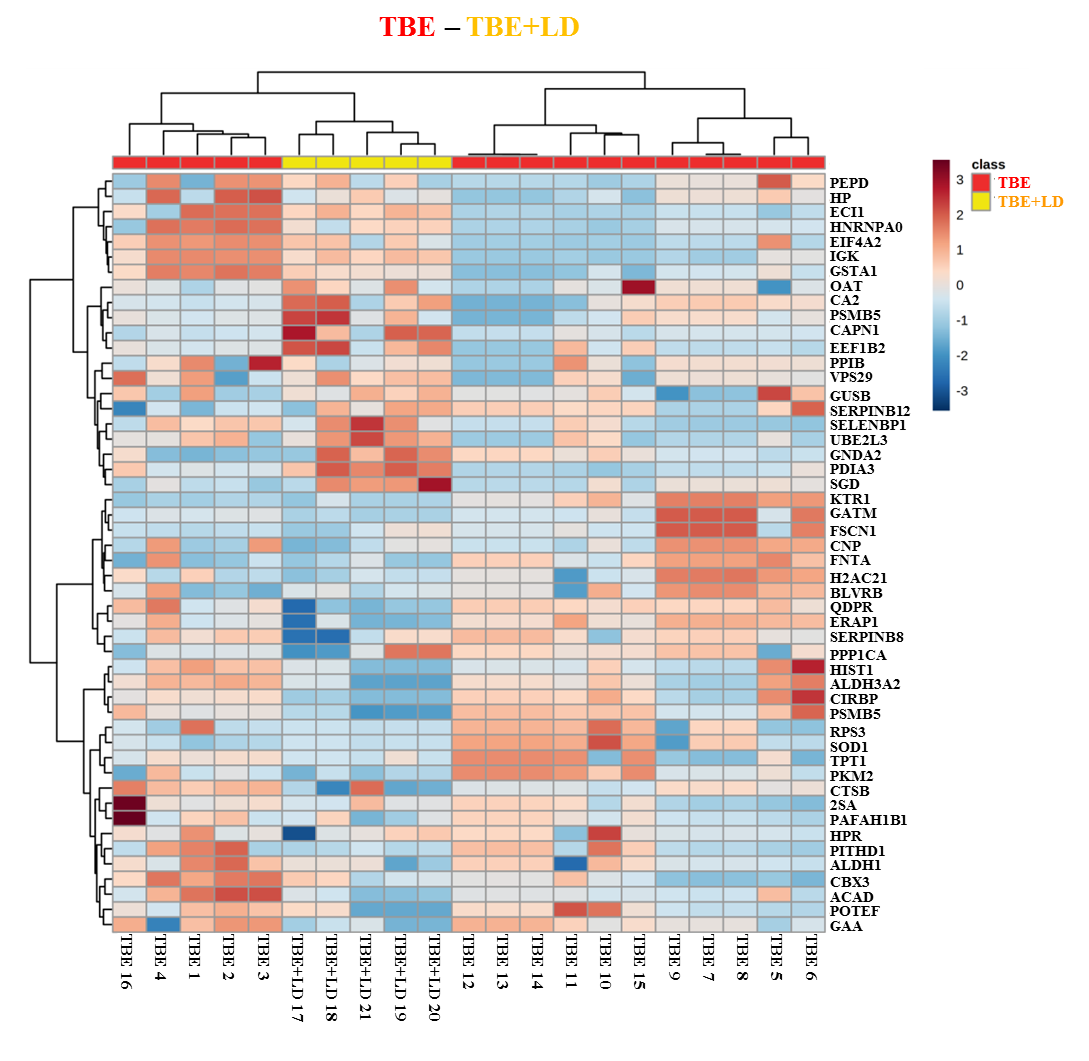

Supplement: Supplementary file 1 [file ijms-23-04374-s001.zip › S4 Fig C.tif]

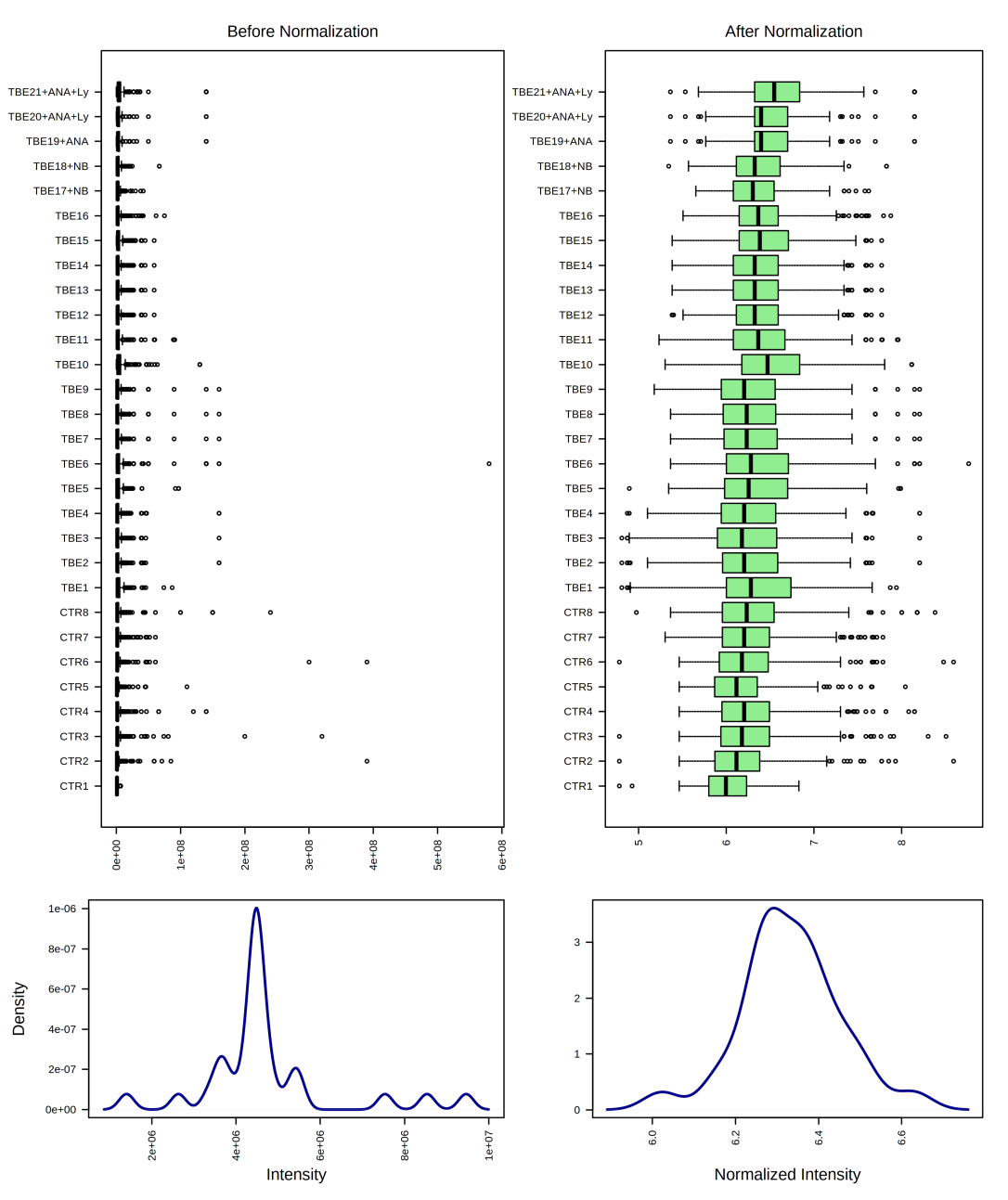

Supplement: Supplementary file 1 [file ijms-23-04374-s001.zip › S5.tif]
